# Optimization of Machine Learning in Various Situations Using ICT-Based TVOC Sensors

**DOI:** 10.3390/mi11121092

**Published:** 2020-12-10

**Authors:** Jae Hyuk Cho, Hayoun Lee

**Affiliations:** School of Electronic Engineering, Soongsil University, Seoul 06978, Korea; leehayoun0916@ssu.ac.kr

**Keywords:** sensory data reliability, measurement applications, prediction, TVOC, LSTM, RNN, GRU

## Abstract

A computational framework using artificial intelligence (AI) has been suggested in numerous fields, such as medicine, robotics, meteorology, and chemistry. The specificity of each AI model and the relationship between data characteristics and ground truth, allowing their guidance according to each situation, has not been given. Since TVOCs (total volatile organic compounds) cause serious harm to human health and plants, the prevention of such damages with a reduction in their occurrence frequency becomes not an optional process but an essential one in manufacturing, as well as for chemical industries and laboratories. In this study, with consideration of the characteristics of the machine learning technique and ICT (information and communications technology), TVOC sensors are explored as a function of grounded data analysis and the selection of machine learning models, determining their performance in real situations. For representative scenarios, considering features from an ICT semiconductor sensor and one targeting TVOC gas, we investigated suitable analysis methods and machine learning models such as LSTM (long short-term memory), GRU (gated recurrent unit), and RNN (recurrent neural network). Detailed factors for these machine learning models with respect to the concentration of TVOC gas in the atmosphere are compared with original sensory data to obtain their accuracy. From this work, we expect to significantly minimize risk in empirical applications, i.e., maintaining homeostasis or predicting abnormal situations to construct an opportune response.

## 1. Introduction

With the development of information and communications technology (ICT) and its applications, data analysis in various industrial fields, especially involving sensory data (i.e., typical time-series data), is gathering great attention [1,2]. By collecting and monitoring sensory data, users and administrators can manage and predict data related to advanced work efficiency, equipment failure, and situation warnings in real time. Legacy sensor systems based on expert experience perform limitedly in extreme or exceptional conditions with a more delayed response than required. Furthermore, recognition accuracy has been a key problem in multiple sensor system studies [3]. In every situation, ICT-based gas sensing data are presented as a jumble of valuable data and noise, requiring analytical methods for their deep comprehension.

The development of ICT technology and the expansion of its applications are accompanied by an increase in both the quality and the quantity of data, while a trend of introducing machine learning technology to sensory data has emerged. In the case of time-series prediction, it is necessary to review regressive models, such as the AR model (autoregressive model), RNN (recurrent neural network), LSTM (long short-term memory), or GRU (gated recurrent unit).

In this study, through a delicate selection of scenarios corresponding to major aspects of ICT TVOC (total volatile organic compound) sensors, a suggestion of analysis methods for each situation, as well as a comparison of the predictive data from RNN, LSTM, and GRU, is performed. The use of this framework of analysis and prediction provides a proper guide for treating and predicting ICT TVOC sensory data to avoid system shutdown and to increase work efficiency through economic processes such as air circulation/ventilation and pollutant removal.

## 2. Materials and Methods

### 2.1. Considerable Features of ICT TVOC Sensors

The data from ICT TVOC sensors can be categorized as semiconductor sensors, ICT sensors, and TVOC gas properties.

#### 2.1.1. Target Gas (TVOC)

Typical toxic gases that pollute the air include monoxide (CO), carbon dioxide (CO_2_), nitrogen dioxide (NO_2_), volatile organic compounds (VOCs), ozone (O_3_), sulfur dioxide (SO_2_), and formaldehyde. In addition, toxic air pollutants include radon and benzene [4]. Among them, VOCs form low-level ozone in the atmosphere through a reaction involving oxygen and sunlight. In the upper atmosphere, O_3_ prevents ultraviolet light from the sun reaching humans. However, ozone is a dangerous compound in the lower atmosphere, causing smog. The concentration of toxic gases in the air, exceeding life-threatening levels, can harm human health and plants, destroying their natural processes [5]. In order to prevent such damages and reduce their frequency of occurrence, it is important to accurately measure the concentration of toxic gases in the atmosphere [6,7].

#### 2.1.2. Semiconductor Gas Sensors

**Figure 1:**
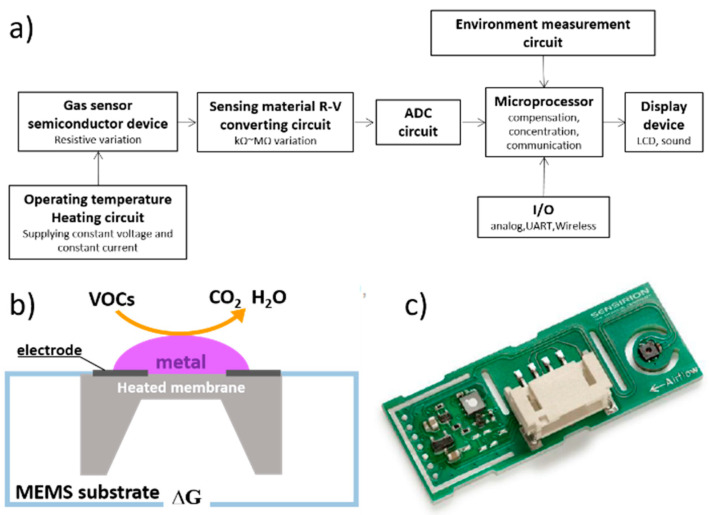
ICT-TVOC sensor: (**a**) semiconductor gas sensor device; (**b**) the principal of the semiconductor sensor; (**c**) SVM 30 (Sensirion). ICT, information and communications technology; TVOC, total volatile organic compounds.

Semiconductor gas sensors function via a change in electrical conductivity when the target gas contacts a ceramic semiconductor surface. This involves an exchange of electrons between the sensing material and the gas upon contacting the sensing material, whereby a reduction–oxidation reaction occurs, providing detection information on the basis of a change in resistance. These sensors react to toxic gas and flammable gas, and the configuration of their detection circuit is simple, allowing simple manufacture and mass production. However, their specificity is hampered as changes in electrical conductivity also occur in response to nontarget gases. Semiconductor gas sensors are often used as TVOC and CO_2_ sensors for measuring indoor air quality [8]. There are two types of semiconductor gas sensors: gas sensors (bulk) and gas modules (gas sensor modules). Semiconductor gas sensors and modules consist of a semiconductor gas sensor element, a heating circuit, a sensing material R–V conversion circuit, an analog-to-digital converter (ADC) circuit, a microprocessor, an ambient environment measurement circuit, a display device, and an I/O circuit. The detailed features of a semiconductor sensor are shown in Figure 1.

The semiconductor gas sensor device consists of a metal oxide gas sensing material such as SnO_2_, WO_3_, and In_2_O_3_, a heater to maintain the proper operating temperature of the metal oxide, and a sensing electrode to measure the resistance of the sensing material. The resistance change of the sensing material of the semiconductor gas sensor needs to be changed in the range of KΩ~MΩ depending on the gas type and gas concentration so an appropriate R-V conversion circuit is required. ADC of 8~12 bit is suitable for semiconductor gas sensor, and ADC with higher resolution can be applied in some cases, and can be applied according to the definition of microprocessor function.

#### 2.1.3. ICT Based Sensors

Information communication technology (ICT) is a varied cluster of digital tools and resources used to communicate, generate, disseminate, store and perform information management [9]. Continuous advancement of miniature environmental sensing devices, microelectronics and communication technologies propelled the development of ICT sensors for being environmentally monitored by its enabled systems and mechanisms while broadcasting timely updates. The effectivity of ICT data does not come from a single expensive sensor but from number of moderate priced sensors distributed within a vast range [10]. ICT sensors produce reliable data by surmounting errors and creating the relation of data from the multi sensors. However, the data obtained by ICT sensors tends to overestimate not only the meaningful values but also the errors.

#### 2.1.4. TVOC Sensor Setup

The sensory data is measured by indoor temperature/humidity, CO_2_ and TVOC. This data is used as training data, composed of outlier and environment information. The experimental set-up to serve as the model is established with three sensor groups, Multi-pixel MOX gas sensor of Sensirion Inc. (model: SVM30). We operated with 100 sensors in a sensor group and extracted representative values of each sensor groups. Within the whole process, temperature and humidity have been measured and controlled. All of the experiment was built with/without the artificial TVOC generating specimen in a small room (16 m^2^) and measured over 24 h. The sensors used were initialized in the open air for 3 h before each experiment [11]. The total data set for this study had have been collected per every second in a month (4 April 2020~7 May 2020), and the training data set (80%, 800 s data per gas) and testing data set (20%, 200 s data per gas) were applied from the entire set. The validation data set was randomly selected from the training data set and applied. Additionally, the scenario temperature/humidity was controlled consistently as 20.4 ± 2.3 °C and 24.5 ± 6.9% RH, the environment in EVENT 1 was controlled as 23.3 ± 1.5 °C and 29.8 ± 6.4% RH, and the one in EVENT 2 was controlled as 24 ± 1.4 °C and 50.1 ± 4.2% RH.

### 2.2. Machine Learning

**Figure 2:**
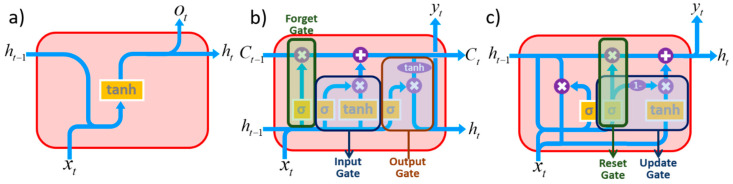
Diagrams of AI models (**a**) recurrent neural network (RNN), (**b**) long short-term memory (LSTM), (**c**) gated recurrent unit (GRU).

In recent years, machine learning has driven advances in many different fields [12,13,14]. The process of predicting the sensory data is used not only for securing reliability of the existing data and defining the cause of faults that had already occurred also in forecasting future to detect the user’s risk in advance. Among the AI models, mainly RNN, LSTM, and GRU are widely used. This tendency is due to the proven stability compared to other AI models [15,16]. These models’ working mechanism is shown as a diagram in Figure 2.

Three of machine learning models have distinctive characteristics on maintaining initial information and working principals. RNN is a type of artificial neural network in which hidden nodes are connected to directed edges to form directed cyclic structure. It is known as a model suitable for processing time series data that appears sequentially, such as voice and text. RNN is composed of repeating chains of neural networks, i.e., output result depends on the previous calculation result. From this characteristic, learning ability of RNN significant decreases as distance between the related information and the point where it is used is far (vanishing gradient problem).

LSTM cells were proposed by Hochreiter and Schmidhuber in 1997, which solved the problem of long-term dependence of RNN cells and rapidly converged learning [17]. The basic characteristic of the LSTM is the storage of information for a long period of process without special treatment. LSTMs, like RNNs, have a chain structure but a different module, containing four interactive gates. In this efficient structure, the initial information is treated with very minor linear operation through the cell state and penetrates the entire LSTM chain so that information continues passing to the next level without significant change. In LSTM, there were three gates: output, input, and erase gate. The GRU removes the output gate from the LSTM, and the role of the input gate and the forward gate are divided to the update gate and reset gate. Usually, it is known that the LSTM is slightly more powerful, but the GRU also shows similar performance in poor computing environment by reducing computational capacity [18].

#### 2.2.1. Detailed Parameters of Machine Learning

- Number of Hidden Layers

A single hidden layer, given enough neurons, can form any mapping needed. In practice, two hidden layers are often used to speed up convergence. A single hidden layer neural networks is capable of universal approximation. The universal approximation theorem states that a feed-forward network, with a single hidden layer, containing a finite number of neurons, can approach continuous functions with mild assumptions on the activation function [19,20]. Problems that require more than two hidden layers were rarely prior to deep learning. Two or fewer layers will often suffice with simple data sets. However, with complex datasets involving time-series or computer vision, additional layers are required.

- Number of neurons

Traditionally, neural networks only had three types of layers: hidden, input and output. Deciding overall neural network architecture, the number of neurons in the hidden layers is a very important part. Both the number of hidden layers and the number of neurons in each of these hidden layers must be carefully considered. The number of neurons in the hidden layers may result in overfitting or underfitting. Additionally, the number of hidden neurons should be between the size of the input layer, the size of the output layer and 2/3 the size of the input layer, plus the size of the output layer, as well as it less than twice the size of the input layer [21].

- Epoch and Batch Size and Iteration

One epoch is when an entire dataset is passed forward and backward through the neural network only once. The number s of epoch is related to how diverse data is. While iteration is the number of batches needed to complete one epoch, the batch size means the total number of training data present in a single batch so it can be called as update frequency. As the size of the training set grows, the accumulated weight changes for batch training become large. This leads batch training to use unreasonably large steps, which in turn leads to unstable learning [22].

#### 2.2.2. Learning by LSTM and RNN and GRU

The continuous time series values are the input; 80% of the total data is used for learning and 20% of values are predicted and compared with real values, following Pareto’s principle [23] in CONSTANT and EVENT 1, however, in EVENT 2, to predict the increasing point, 50% of the values are predicted and used for learning to predict the sudden increasing point. The number of hidden neurons is fixed as 15, and epoch is fixed as 13, these values do not significantly affect the result, so the fixed value is used.

**Figure 3:**
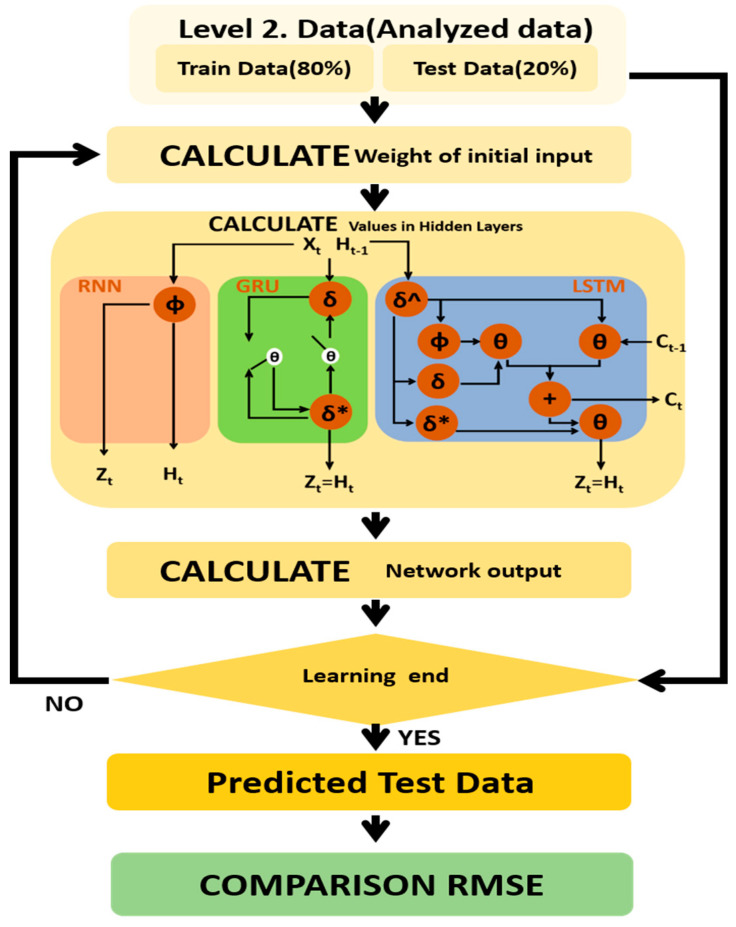
AI prediction workflow as δ: input, δ*: output, δ^: forget gate, Φ: input modulation.

The details of the machine learning to be considered in the above workflow are the number of hidden layers and the number of neurons, governing learning efficiency. According to principle of number of neurons, it is set as 15 while the number of neurons in the input fixed to 30 and output layer fixed to 1 (involved with the input and output data). Whereas the number of hidden layers is studied 0, 2, 9, 11 to optimize the accuracy of learning. The workflow is shown in Figure 3.

### 2.3. EDA (Exploratory Data Analysis)

Previously arithmetic EDA is widely pertained [24,25], but now it needs to apply machine learning for developing sophisticated data analysis skill. Among the well-known methods, there are only a few securing accuracy.

#### 2.3.1. K-Means

The K-means algorism is one of clustering methods, presented as assigning objects to the nearest cluster by distance. Assigning each observation to the cluster is with the centroid and the least squared Euclidean distance, set as cost function. Thus, it makes the cluster by updating the groups to minimize this cost function. K-means algorithm does have limitations; sensitivity on outliers. During the updating process, the outliers may cause serious distortion of the total mean—the centroid is not located on the real center of the group but located on a nearby outlier. For elimination of the outliers, the Z-score or using the k-medoids algorithm prevents outlier problem [26].

#### 2.3.2. Z-Score

The Z-score is a numerical value which makes statistically normal distribution and shows the position of each data on the standard deviation. The standard value indicates how far the original value is from the average, as a negative value implies below the average and a positive value implies above the average.

#### 2.3.3. Possibilistic Fuzzy C-Means (PFCM) Clustering Method for Analyzing Sensory Data

In general, many studies have been conducted on fuzzy clustering to classify patterns and the fuzzy C-means (FCM) algorithm has been used most frequently [27,28]. In order to solve the problems of the FCM method, possibilistic C-means (PCM) clustering algorithm is proposed to allocate typicality as determining an absolute distance between a pattern and a center value. However, the PCM algorithm also has a problem that the clustering result is sensitively reacted according to the initial parameter value. To solve this problem, the PFCM algorithm, combining FCM and PCM by the weight sum, has been studied [29].

## 3. Results

**Figure 4:**
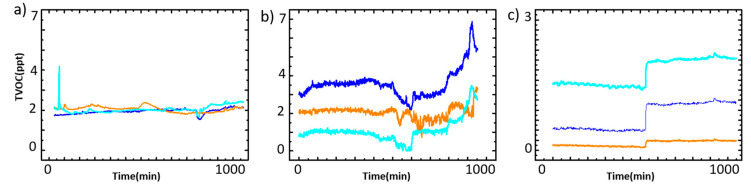
Raw data from the TVOC sensor groups: (**a**) CONSTANT situation, (**b**) EVENT 1: increasing TVOC rate at 1.45 ppt/min, (**c**) EVENT 2: increasing TVOC rate at 10.02 ppt/min. Each line color indicates each sensor.

The three scenarios are made from the long-term observation of real indoor TVOC sensing: in ordinary situations (CONSTANT), with the gas concentration increasing relatively within a long period (EVENT 1), and with a sudden shock of gas concentration (EVENT 2). The training sets for each machine learning model are provided according to the machine learning architecture, where RNN takes the only difference of the situation, the TVOC increasing rate, as in EVENT 1, where the average of the TVOC increasing speed is 1.45 ppm/min, and in EVENT 2, at 10.02 ppm/min. The CONSTANT has no significant TVOC concentration changes as shown in Figure 4.

In the CONSTANT situation there is a sudden change that appears for 2–3 s and then disappears. This sudden change is considered as an outlier and the data immediately before and immediately after the outlier data tends to show generally similar values. This is a type of outlier caused by an error occurring in driving the semiconductor sensor.

The semiconductor sensor reads a minute difference in resistance as a signal from the oxidation/reduction reactions occurring on the surface of the semiconductor. The signal generated on the semiconductor surface indicates an abnormal value due to various causes. The reason that the data before and after outlier shows the same value is because the resistance signal is recovered immediately after the abnormal value and shows the normal value again. On the other hand, the increasing tendency in the EVENT 1 situation is the data feature for recognizing the situation required for IoT sensors, as the indoor IoT sensors acquire the data representing a certain space of information such as the above increasing situation. On the contrary, the sudden increasing gas concentration in EVENT 2 situation is not often seen in other IoT gas sensors. This is due to the volatility, a singularity of TVOCs, because when the source is inserted into the system, the concentration of TVOCs in the air can increase rapidly to more than 10 ppm/min.Therefore, each scenario represents the characteristic of ICT-TVOC semiconductor sensors as shown in Table 1 as we designed.

### 3.1. Failure Sensor Determination

In order to exclude sensors, the element of the sensor was tested separately to verify the fault of the sensor. The detailed process and results are as follows.

**Figure 5:**
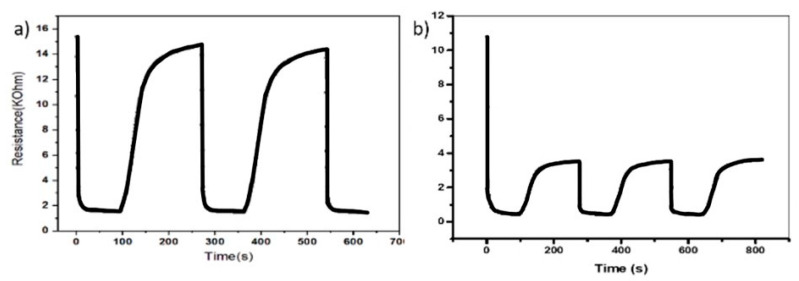
Result of sensor reliability test: (**a**) resistance of sensing element changes with an injection of 1 ppm of acetone, (**b**) resistance of the sensing element change with an injection of 10 ppm of acetone.

As shown in Figure 5, the result of injecting acetone at 1 and 10 ppm, the source of the sensor reaction, into each sensor three times, one sensor (marked as orange line in Figure 4) shows abnormal behavior, a greater resistance changes in the 1 ppm injection than in 10 ppm. It was judged as a malfunction. From this basic surrounding data, it was obviously found that the atmosphere, especially the humidity, changes may cause TVOC concentration changes [30], so that it must be heavily considered. Incidentally, as observed during the experiment, the phenomenon whereby a specific sensor does not reach enough sensitivity as the others is one common drawback of the ICT sensors, because these are a combination of not secure and inexpensive sensors. Thus, the data of the defective sensor was entirely removed.

### 3.2. Analysis

#### EDA

In this study, K-means, Z-score, and PFCM are considered as analysis tools for raw data treatment before machine learning. It is a necessary procedure to increase accuracy of prediction. The TVOC sensory data in the CONSTANT situation without external environmental changes mostly, the main cause of error is occurred by the structural characteristics of the semiconductor sensor, the hypersensitive reaction of device, caused by the influence of minor changes of temperature and humidity, which is irrelevant to the reflection (high/low) of TVOC concentration. Therefore, in the CONSTANT situation, the 3D Z-score method (temperature–humidity–TVOC) is mandatory to select valid data and eliminate outliers. However, apart from these features of the TVOC sensing hardware, TVOC itself also causes specific characteristics, i.e., its typical volatility is affected by temperature and humidity. In TVOC increasing circumstances (EVENT 1, EVENT 2), the influence of temperature and humidity may cause the TVOC changes so that outliers from these atmosphere data may not be eliminated by using Z-score. In all situations, normalization is preceded and a selective Z-score is performed for outlier elimination (Level 1). Then, K-means and PFCM carry out clustering. As the pick points of TVOC in EVENT 1 and 2 are considered as errors in the analysis tools, we performed partial-analysis, within a constant behavior part in each situation.

The Z-score is a value that statistically creates a normal distribution and shows where each data is located on the standard deviation. The standard value indicates how far away the data is from the mean—negative, below the average; positive, above the average.

Over the standard score of 0.0 (deviation value: 50) is 50% of the total and over the standard score of 2.0 (deviation value = 70) is 5.275% of the total. However, deleting 5.275% of data from the original one cannot reserve enough train data for machine learning. Thus, it needed to apply a better algorithm to define the outliers.

**Figure 6:**
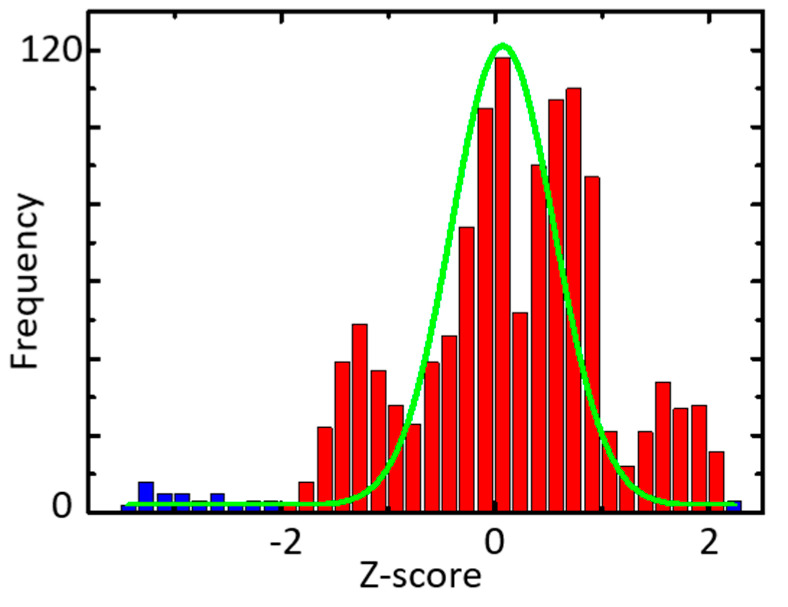
Histogram example of Z- score in COSTANT.

**Figure 7:**
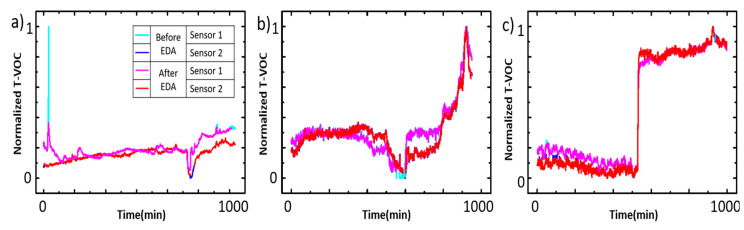
Analysis results in normalized scale. (**a**) CONSTANT, (**b**) EVENT 1, (**c**) EVENT 2.

For example, as shown in Figure 6, in CONSTANT-Sensor 1 (blue bar in Figure 4), the histogram of the Z-score shows 3.07% outliers, centering on the PFCM, as shown in Table 2. This process was repeated on each sensor in every situation, as shown in Figure 7, while K-means or PFCM was chosen depending on the accuracy of the clustering.

**Figure 8:**
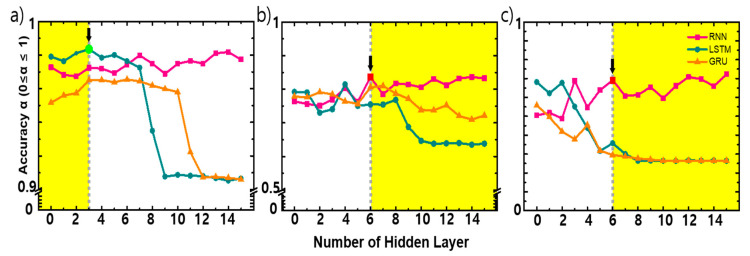
Accuracy result of the prediction of number of hidden layers: (**a**) CONSTANT, (**b**) EVENT 1, (**c**) EVENT 2. The yellow region indicates the optimized number of hidden layers of each situation. To save computation resources, the marked (with arrow) number of hidden layers are recommended.

As shown in Figure 8, with an increasing number of hidden layers, the accuracy changes. Depending on the data pattern, the tendency of the accuracy of each machine learning model is different. As shown in Figure 8a, in the CONSTANT situation, LSTM shows a better performance with less than three hidden layers even though the difference is quite minor (0.9 ≤ σ ≤ 1). In direct contrast, as shown in Figure 8b,c, in EVENT situations, LSTM and GRU show poor performance as increasing the number of hidden layers and RNN produces better accuracy with more than 6 hidden layers.

## 4. Discussion

The previous studies have shown that data analysis must be performed for pre-processing the machine learning but there is a lack of clear technical description. It appears that data is being processed through a traditional arithmetic method. Because most of the actual data will be lost or distorted unless the proper method is introduced according to given data pattern, the careful approach on the data preprocess is required. Machine learning has been studied as its use has emerged in multiple fields, such as meteorology, geometry, medical science, etc.—mostly dealing with time-series data. However, in previous studies, models are sporadically chosen without the prior consideration of criteria and detailed parameters which should be described.

In this paper, three representative detailed scenarios are presented, derived from the analysis of three groups of sensors and machine learning methods to provide optimized information of sensor data. Further, from the previous simple collection of ICT sensor data, detailed parameters of applied machine guidance were derived for sensor data analysis and prediction. Additionally, detailed context is provided of the appropriate criteria for each situation/model. Data of this paper is not relative to resampling to calculate variability, whereas the focus is on analyzing trends (situations) using TVOC gas data generated in existing indoor situations and suggesting prediction methodologies for each situation. Therefore, it was randomly selected from the total set and applied to the training set and test set. However, the results on validation might over-simplify the problem, and a cross-validation approach is needed to be applied in future study. However, the verification set approach may have a changeable problem depending on whether a specific value is included in the verification set, and a cross-validation approach supplemented with this may be applied in future studies. It is also necessary to develop/verify detailed algorithms that verify the reliability of additional sensor data, such as the decreasing pattern of the sensor target and the criteria of the outlier as well as various sensor data required to consider the association analysis between data in various environments/situation.

## 5. Conclusions

TVOC sensing shows the typical features of the ICT semiconductor gas sensing trend. In a variable TVOC situation, each feature of the sensor causes different types of errors, for example, the characteristics of the ICT sensor causes a difference of measurement sensitivity or reactivity between units, while the TVOC itself also causes sensing errors with minor humidity changes. To overcome these defects and replicate the reality, relatively reliable sensors with high market share (Multi-pixel MOX gas sensor of Sensirion Inc., Stäfa, Switzerland), are used in this study and the experiments are performed repeatedly. In addition, an experimental method for determining abnormal behavior of each sensor is presented. During the treatment of data process, dependence on performance and difficulty of acquiring enough training data can be faced. In particular, in the case of using deep learning, sufficient learning data is required to obtain an efficient result. Therefore, for novel research, it is necessary to choose various methods or increase the training data according to the amount of data. Selective exploratory data analysis (EDA) methods, such as K-means and PFCM, can be used according to the circumstances and to extract refined data from the raw data including noise. This process leads to better effectivity if you do data predictions before.

The suggested EDA methods retain more valid data than other noise removal methods, so that more training data is secured, which leads higher prediction accuracy. The accuracy difference in machine learning models do not indicate the superiority of certain methods, so it is requisite to introduce proper methods to each situation. This tendency occurs with RNN’s cyclical structure and lower retention capacity for long-term data than LSTM. In the TVOC environment, these three models can be applied in different situations. Theoretically, RNNs can handle long-term dependencies perfectly. In fact, for simple examples, RNN can solve the problem by carefully choosing parameters. However, in most of the cases, RNNs do not solve the long-term dependence problems. Remembering information for a long period of time is the basic behavior of this model, so there is essentially no possibility of a period dependence problem caused by the parameters of the model. On the other hand, LSTM was designed explicitly to avoid the problem of long dependency. In an EVENT situation where the state of the previous state does not affect the next state, RNN without long-term dependencies is rather helpful for perfect calculation, while in the CONSTANT situation where the previous state affects the next state, the above problem of the RNN has fatal consequences. Ultimately, the EVENT situations, such as sudden increase or decrease of target gas concentration, RNN serves a purpose properly by using long-term data for pattern learning, then disappears, and short-term data affects prediction. In contrast, LSTM and GRU performs to predict future values with short/long term memory to reflect overall situation when there is need to maintain a constant TVOC level.

Not only the type of model is relevant to prediction of TVOC, but also the detailed factors of the machine learning, which cause serious changes of accuracy. Meticulous consideration of hidden layers numbers is required since a moderated number of hidden layers is the key factor of fast and accurate prediction.

Previously, it has been shown empirically that the best performance of a neural network is when the number of hidden nodes is log (T), where T is the number of training samples [31]. This principal is also partly observed in this study in whole situations, LSTM and GRU give better performance with less than three hidden layers. However, RNN shows comparatively stable accuracy with change (increase) of number of hidden layers and miner linearity is shown.

**Figure 9:**
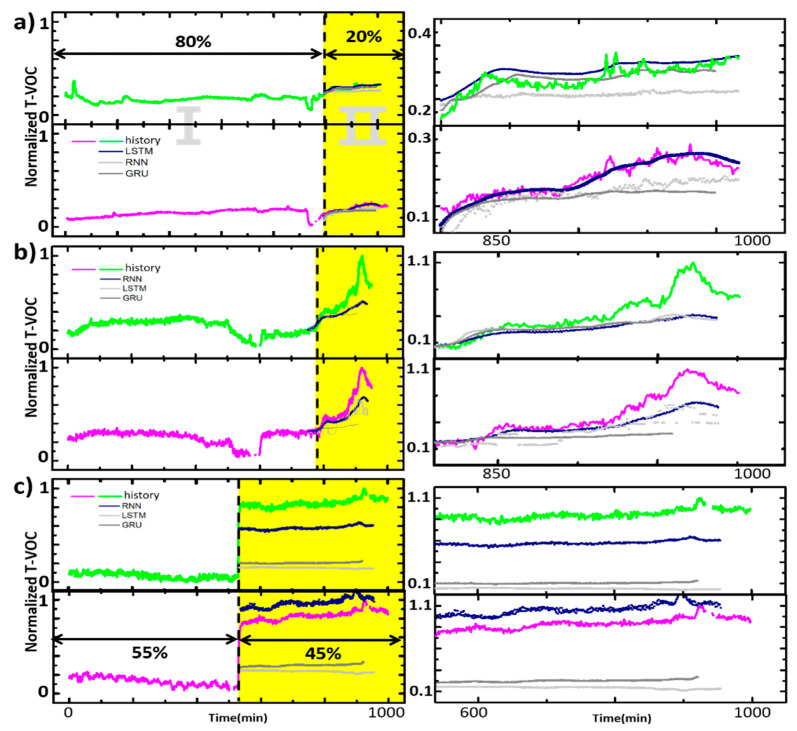
Two sensor data and prediction result on each situation. Region Ⅰ: train data, Region Ⅱ: predicted data. Blue line indicates the best prediction method for each situation. It is as (**a**) CONSTANT situation: LSTM (HL:3), while RNN (HL:15)—right gray, GRU (HL:15)—gray, (**b**) EVENT 1 situation: RNN (HL:6) while LSTM (HL:15)—right gray, GRU (HL:15)—gray, (**c**) EVENT 1 situation: RNN (HL:6) while LSTM (HL:15)—right gray, GRU (HL:15)—gray.

The best condition in each situation is as Table 3. The comparison of machine learning models with optimized hidden layers is shown in Figure 9. The EDA method was determined by the approach of clustering, the CONSTANT situation is treated with 3D Z-score and PFCM, and the EVENT situation with partial Z-score and K-mean. To compare the best method and the others, only the best performing number of hidden layers on each method is selected as comparative group.

As discussed in this paper, we are going to develop real-time data acquisition algorithms that utilize the optimization of data selection, analysis, and machine learning techniques according to the environment patterns in which sensory data is obtained.

The machine learning process suggested in this study can be used as follows. After classifying the input data into CONSTANT, EVENT1 and, EVENT2, a solution suitable for each situation is provide.

CONSTANT: As a pre-processing for prediction, the outliers in the test data are removed before performing prediction, and the difference in prediction accuracy between the test data with/without the outlier removal the outliers is calculated to extract the prediction errors.
EVENT 1: Through this process the peak point of gas concentration and its arrival time to the peak of the gradually increasing pollutant gas in the general situation of pollution is predicted with prediction accuracy.
EVENT 2: In the case of a very rapid increase in polluted gas, the maximum concentration of polluted gas is predicted and induce suitable the level of response (ventilation, evacuation, etc.).

## Figures and Tables

**Table 1:** Presentative feature on the scenarios. Data from the CONSTANT situation is showing a typical semiconductor sensor data feature, while in EVENT 1 and EVENT 2, the ICT sensor and TVOC gas characteristics are relatively displayed.

Scenario	CONSTANT	EVENT 1	EVENT 2
Feature	Semiconductor	ICT	TVOC

**Table 2:** **Level 1:** Analysis process of normalization (min–max scaler) and outlier elimination (3D Z-score/Z-score). **Level 2:** The clustering method (K-means/PFCM) and accuracy of clustering (0 ≤ Ac ≤ 1, calculated from the average of the distance between each sensory data and the clustering center created by PFCM and K-means)**.**

Level	CONSTANT	EVENT 1	EVENT 2
Level 1	Min-max scaler		
	3D Z- Score	Partial-Z score	
Level 2	PFCM	K-means	
	0.07	0.26	0.55

**Table 3:** Condition for the best performance under each situation.

Method	Level	COSTANT	EVENT 1	EVENT 2
EDA	Level 1	3D Z- Score	Partial-Z score	
	Level 2	PFCM	K-means	
Machine learning	Model	LSTM	RNN	RNN
	HL *	<3	6<	6<

* HL: number of hidden layers.
